# Antimicrobial mechanism of *Phyllanthus niruri* L. against oral pathogens: a scanning and transmission electron microscopy study

**DOI:** 10.3389/fdmed.2026.1696836

**Published:** 2026-03-02

**Authors:** Vanishree H. Shivakumar, Anand S. Tegginamani, Raghavendra M. Shetty, Annapurny Venkiteswaran, Nurhayati Mohamad Zain, Nurul ‘Izzah Mohd Sarmin, Zolkapli Eshak, Eddy Hasrul Hassan

**Affiliations:** 1 Faculty of Dentistry, SEGi University, Kota Damansara, Malaysia; 2 College of Dentistry, Ajman University, Ajman, United Arab Emirates; 3 Faculty of Dentistry, Universiti Teknologi MARA, Sungai Buloh, Malaysia; 4 Atta-ur-Rahman Institute for Natural Product Discovery, Universiti Teknologi MARA, Puncak Alam, Malaysia; 5 Faculty of Pharmacy, Universiti Teknologi MARA, Puncak Alam, Malaysia

**Keywords:** cell membrane, *Enterococcus faecalis*, microscopy, *Phyllanthus niruri*, plant extracts

## Abstract

**Introduction:**

Endodontic infection in primary teeth is a multimicrobial disease involving a rich combination of bacterial species. The secondary metabolites from the medicinal plant *Phyllanthus niruri* L. have antimicrobial properties. This *in vitro* research evaluated the antimicrobial activity of *P. niruri* and its mode of action against *Enterococcus faecalis*, *Porphyromonas gingivalis*, and *Treponema denticola*.

**Methodology:**

The antimicrobial activities of an ethanolic extract of *P. niruri* and a triple antibiotic paste (TAP) as a positive control were evaluated using the disc diffusion method, and their minimum inhibitory concentration (MIC) and minimum bacterial concentration were determined. Scanning electron microscopy (SEM) and transmission electron microscopy (TEM) were used to observe the cellular damage induced by the extract. A one-way ANOVA followed by Tukey's *post hoc* test was used to compare the mean zone of inhibition for all three microbes. The level of significance was set at *P* < 0.05.

**Results:**

The herbal extract of *P. niruri* had the highest inhibitory effects against *T. denticola* (16.06 ± 3.13), followed by *P. gingivalis* (14.54 ± 2.28 mm), and *E. faecalis* (10.10 ± 0.71 mm). However, it was less than the TAP (*p* < 0.001). *P. niruri* exhibited bacteriostatic potential with its MIC against all microbes. Furthermore, SEM and TEM found severe membrane deformation and extensive cytoplasmic leakage in *T. denticola* and *P. gingivalis* compared to *E. faecalis*.

**Conclusion:**

Based on the study results, a 250 mg/mL concentration of *P. niruri* could be beneficial as a potential medicament for reducing root canal pathogens.

## 1 Introduction

Despite advances in understanding the prevalence, etiology, prevention methods, and less-invasive management of the carious process, the disease and its effects remain an important clinical issue in pediatric dentistry (1). Microbes are the most common cause of dental caries, which further invade the root canal space with their toxins through the dentinal tubules. These infected root canals are a prevalent concern in both primary and permanent dentition, as they may lead to severe pain, swelling, abscesses, and even tooth loss (2). In primary teeth, endodontic infection is a multimicrobial disease with a rich combination of bacterial species. From the root canal of a symptomatic primary tooth with periapical pathology, both aerobic and anaerobic microorganisms have been cultured and identified (3). Among these species, *Prevotella intermedia*, *Lactobacillus acidophilus*, *Streptococcus mutans*, and *Streptococcus sobrinus* have been linked to the initial stages of dental caries and pulpal surface colonization (4). Similarly, other studies have demonstrated the presence of both aerobic and anaerobic species in endodontic infections (5, 6). Cogulu et al. reported some of the infectious bacteria in deciduous and permanent teeth that were associated with both endodontic and periapical signs and symptoms (7). Microorganisms such as *Enterococcus faecalis*, *Porphyromonas gingivalis*, and *Treponema denticola* are significantly associated with recurrent endodontic infections and treatment failures. *E. faecalis* is a facultative anaerobe and is widely recognized in systematic research as a resistant bacterium. It is frequently isolated from cases of endodontic treatment failure due to its ability to survive in nutrient-deprived environments, penetrate dentinal tubules, and resist conventional intracanal medicaments (8). Conversely, the obligate anaerobes *P. gingivalis* and *T. denticola* play significant roles in biofilm formation, swelling, tenderness upon percussion, sinus tract development, and periapical abscesses (9). These particular microbes were chosen in this study to provide a clinically relevant microbial model for assessing the antimicrobial effects of the plant extract against root canal pathogens. They also represent both persistent facultative anaerobic pathogens and highly virulent obligate anaerobes. The primary goal of endodontic treatment is to remove the infection from the root canal. Conventional treatment approaches to remove these bacterial infections include performing vital and non-vital pulp therapies using several materials (10). Currently, the non-vital pulp therapies for primary teeth are pulpectomy and lesion sterilization tissue repair (LSTR). The overall efficacy of pulpectomy and LSTR is primarily determined by clinical and radiographic evaluations of the tooth, instrumentation procedures, removal of the smear layer, irrigation, and the quality of the obturation process using appropriate materials (11). However, these conventional endodontic treatments do not allow for the complete removal of these diverse bacteria from infected root canals due to the complexity of the root canal morphology. In addition to this, it is sometimes impossible to gain the appropriate access to the root canals. This may be due to children's uncooperativeness and small mouth opening (12). LSTR, a procedure that uses a triple antibiotic paste (TAP), is a non-invasive, less time-consuming treatment option for infected deciduous teeth with compromised pulpectomy. A mixture of antibiotics, such as metronidazole 400 mg, ciprofloxacin 200 mg, and minocycline 100 mg in the ratio of 1:1:1, can sterilize infected root dentin with necrotic pulp in deciduous teeth. The local use of a triple antibiotic paste may have other unintended consequences. Because of its radiolucent appearance on radiographs, judging the filling's quality is difficult, and discoloration may occur. Other drawbacks include pharmacological side effects, allergic responses, the possibility of developmental defects in permanent teeth if used in primary teeth, and the chance of developing antibiotic-resistant bacterial strains. However, to date, there is no data to prove which medicament is more effective than the other (13). Therefore, the need for alternative therapeutic agents made from plants as a natural remedy in dental care has grown to address the drawbacks of the current medications and replace these pulpal medications for endodontic therapy. In the literature, several plants have been proposed as potential sources of innovative endodontic treatments. In dentistry, they have been employed as an analgesic, sedative, anti-inflammatory, antibacterial, and endodontic irrigant (14). As a natural remedy, traditional medicinal plant extracts have recently gained popularity in endodontics (15). Among them, *Phyllanthus niruri* (*Phyllanthaceae* family) is widely used in Ayurveda against various microbial infections. It is a prominent medicinal plant, and its leaves have been used to treat a variety of gastrointestinal conditions. It has been reported to have anti-inflammatory, hypoglycemic, antiviral, antioxidant, antibacterial, antifungal, immunomodulatory, antidiabetic, antiplasmodial, anticancer, anticarcinogenic, hepatoprotective, and hyperlipidemic properties. Terpenoids, flavonoids, proteins, phenols, quinones, glycosides, alkaloids, hypophyllanthin, lignans, arabinogalactan, phyllanthin, amino acids, carbohydrates, epicatechin, gallocatechin, gallic acid, and epigallocatechin are among the many secondary metabolites that are abundant in the plant (16, 17).

To date, to the best of our knowledge, no research has been conducted to assess the antimicrobial mechanism of action of *P. niruri* on the structural and deeper cellular damage caused by root canal pathogens. Therefore, by considering its widely recognized medicinal value, this investigation was designed to evaluate the antimicrobial effect of *P. niruri* L. on the surface structural alterations and deeper cellular damage caused by *E. faecalis*, *P. gingivalis*, and *T. denticola* using a scanning electron microscope and a transmission electron microscope.

## 2 Materials and methods

The experiment was conducted using an *in vitro* experimental study design over 6 months in a laboratory at Universiti Teknologi MARA. The ethics committee of the institution approved the study [REC/10/2022 (PG/MR/251)]. This study employed a controlled experimental paradigm to evaluate the antimicrobial efficacy of *P. niruri*. Hypothesis testing was conducted by comparing treated samples against negative and positive controls. The conceptual design integrated quantitative data [minimum inhibitory concentration (MIC) and minimum bacterial concentration (MBC)] with qualitative ultrastructural analysis [scanning electron microscopy (SEM) and transmission electron microscopy (TEM)] to triangulate the mechanism of action.

### 2.1 Culturing of microorganisms

Three bacterial strains of *E. faecalis* 51,299, *P. gingivalis* 33,277, and *T. denticola* 35,405 were purchased from the Deutsche Sammlung von Mikroorganismen und Zellkulturen in Germany. *E. faecalis* was cultivated on Brain Heart Infusion (BHI) agar and incubated at 37°C for 24 h, while *P. gingivalis* and *T. denticola* were cultured in an anaerobic chamber and incubated at 37°C for 48 h with a gas pack. A final inoculum of 1.5 × 108 CFU/mL was obtained by modifying the turbidity of an overnight culture of all the microorganisms to 0.5 McFarland standards to create a standard bacterial suspension.

### 2.2 Preparation for positive control

Three separate tablets were blended to form a TAP, which served as a positive control in the experiment. First, the coating was removed from the pills that contained doxycycline (100 mg, Duopharma Biotech Berhad, Malaysia), metronidazole (Axcel® 400 mg, Kotra Pharma (M) Sdn Bhd, Malaysia), and ciprofloxacin (Ciprodac® 200 mg, Cadila Pharmaceuticals Ltd., India). The tablets were then ground into a powder using a sterile mortar and pestle. All the powdered antibiotics were mixed uniformly in a 1:1:1 ratio with sterile water to obtain a final concentration of 1.0 mg/mL.

### 2.3 Preparation of ethanolic extract of *P. niruri* leaves

To identify the species, mature leaves of the *P. niruri* plant were obtained from a nursery and sent to the Herbarium, Universiti Kebangsaan Malaysia. The species' confirmation was obtained along with the following corresponding voucher number: ID066/2023. Then, 50 g of powdered *P. niruri* leaves was combined with 500 mL of 99.8% ethanol. The ethanolic extract of *P. niruri* plant leaves was created using Troung et al.’s technique with a few minor adjustments (18).

### 2.4 Disc diffusion assay

**Figure 1:**
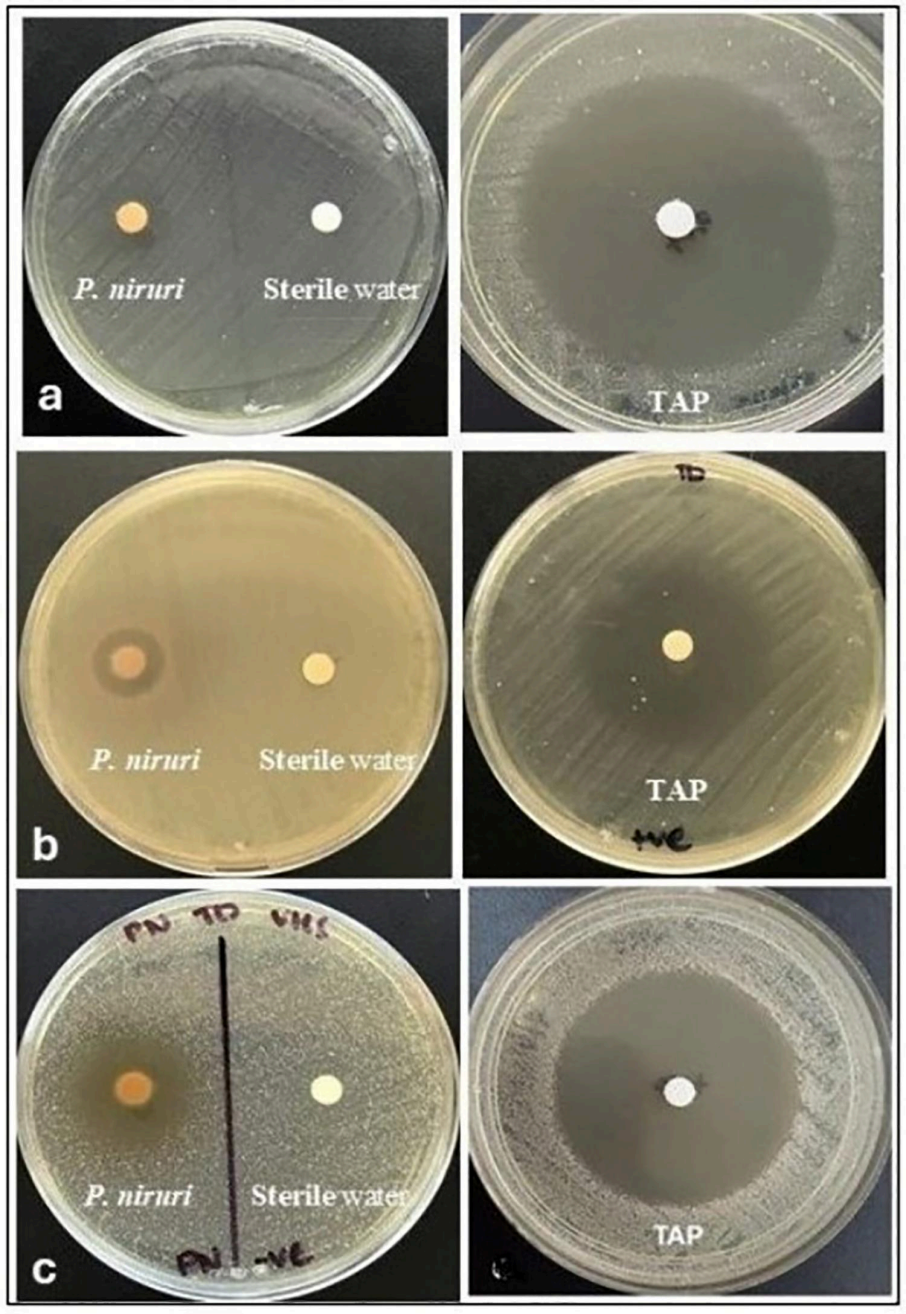
**(a–c)** The antibacterial activity of *P. niruri* and the TAP against *E. faecalis, P. gingivalis*, *and T. denticola, respectively*. Sterile water was used as a negative control.

The antibacterial properties of the herbal plant extract were investigated using the Kirby–Bauer disc diffusion technique (19). Dimethyl sulfoxide (DMSO) and sterile distilled water were blended with the extract to reach a concentration of 250 mg/mL. The final DMSO concentration was kept below 1% and then passed through a Millipore filter. Subsequently, blank antimicrobial susceptibility discs (OxoidTM, Thermo Fisher Scientific, USA) with a 6 mm diameter were loaded with 20 μL of *P. niruri* leaf extract. The positive and negative control groups were TAP and a disc of sterile water, respectively. Each agar plate's surface was uniformly coated with the standard bacterial suspension. The agar plates were then covered with discs that contained the plant extract and the negative and positive controls. All the agar plates with the discs were kept in the incubator. Plates with *E. faecalis* were kept in the incubator at 37°C for 24 h. Agar plates with *P. gingivalis* and *T. denticola* were kept in a CO_2_ incubator at 37°C along with a gas pack for 48 h. Following the incubation period, a digital caliper was used to measure the zone of inhibition (ZOI) in millimeters (Figures 1A–C).

### 2.5 Minimum inhibition concentration

Using the microdilution method, the MIC of *P. niruri* against *E. faecalis*, *P. gingivalis*, and *T. denticola* was established. Up to 10 wells, each with 100 μL of BHI broth, were arranged in rows A, B, and C of the 96-well plate. The first well in each row was then filled with 100 μL of plant extract (starting at 500 mg/mL). A final concentration range of 0.49–250 mg/mL was achieved through 2-fold serial dilutions from well 1 to well 10. Each well was subsequently supplemented with 10 μL of bacterial culture. As a blank control in row E, the extract was serially diluted in bacteria-free broth. Sterility control was ensured by duplicate wells containing BHI broth alone, while growth control was provided by triplicate wells with BHI broth containing bacteria. In addition, the positive control consisted of duplicate wells that contained the TAP and bacteria. After the 2-fold serial dilution process, *E. faecalis* plates were incubated for 24 h at 37°C. For 48 h, *P. gingivalis* and *T. denticola* plates were maintained at 37°C in a CO₂ incubator with a gas pack. The optical density (OD) of the wells was measured at 600 nm using a microplate reader after incubation. The minimal extract concentration was determined by comparing the experimental group's OD value to that of the blank control group, with a difference of less than 0.05 considered significant (20).

### 2.6 Minimum bacterial concentration

The determination of the MBC was performed after the MIC assessment. Accordingly, 10 mL was collected from the wells of 96-well plates preceding the extract's MIC and inoculated into BHI-agar plates. Next, the plates were incubated based on the respective microbe. Incubation of *E. faecalis* was conducted in the incubator at 37°C for 24 h. *P. gingivalis* and *T. denticola* were incubated in a CO_2_ incubator at 37°C along with a gas pack for 48 h. After incubation, the formation of colonies was observed. The MBC refers to the lowest concentration of the extract where no bacterial colonies are detected on the agar plate, suggesting complete bacterial eradication rather than just inhibition (20).

### 2.7 Preparation and fixation of samples for SEM evaluation

Sterile glass slides were marked for orientation and placed into labeled wells of a six-well microtiter plate, assigned as control, BHI with plant extract, and BHI with sucrose and plant extract. Each well received 5 mL of bacterial inoculum, with 80 µL replaced by plant extract in treatment groups to reach the desired concentration. The plates were incubated to allow bacterial growth and interaction with the extract. After incubation, the slides were washed with 1 mL of phosphate buffered saline (PBS) and transferred to a new plate containing 5 mL of 2.5% glutaraldehyde for fixation. The fixed samples were sealed and submitted for SEM analysis (Model: FEI Quanta 450 FEG, Thermo Fisher Scientific, Waltham, MA, USA) (21).

### 2.8 Preparation and fixation of samples for transmission electron microscopy evaluation

Four sterile Falcon tubes were prepared for two control and two experimental groups treated with plant extracts. Each tube received 5 mL of bacterial inoculum, with 80 µL replaced by plant extract at its MIC for the experimental groups. *S. mutans* and *E. faecalis* were incubated at 37°C for 24 h, while *P. gingivalis* and *T. denticola* were incubated at 37°C for 48 h in a CO₂ incubator. After incubation, the samples were centrifuged at 24 rpm for 10 min. The pellets were washed with PBS, recentrifuged, and fixed with 5 mL of 2.5% glutaraldehyde. The tubes were sealed and submitted for TEM analysis (Model: FEI Tecnai G2 Spirit BioTwin, Thermo Fisher Scientific, Waltham, MA, USA) (21).

### 2.9 Statistical analysis

The Statistical Package for Social Sciences for Windows, version 22.0 (IBM Corp, Armonk, NY, USA), was used to perform the statistical analyses. One-way ANOVA was used to compare the mean zone of inhibition of the *P. niruri* herbal extract for different bacterial species. The independent Student’s *t*-test was used to compare the mean zone of inhibition against different organisms between the extract and the triple antibiotic paste. The level of significance was set at *P* < 0.05.

## 3 Results

Table 1 presents a comparison of the mean zone of inhibition for different bacterial species using the herbal *P. niruri* extract. For *E. faecalis*, the mean ZOI was 10.10 ± 0.71 mm, with values ranging from 9.3 to 11.5 mm. For *P. gingivalis*, it exhibited a mean ZOI of 14.54 ± 2.28 mm, with values ranging from 11.0 to 16.7 mm. For *T. denticola*, it had a mean ZOI of 16.06 ± 3.13 mm, with values ranging from 11.8 to 20.5 mm. The test results indicated statistically significant differences between the mean ZOIs for all three bacterial species when using the *P. niruri* herbal extract, at *p* < 0.001.

**Table 1:** Comparison of the mean zone of inhibition of the *P. niruri* herbal extract for different bacterial species using one-way ANOVA.

Bacterium	*N*	Mean	SD	Min	Max	*p*-Value
*E. faecalis*	9	10.10	0.71	9.3	11.5	<0.001*
*P. gingivalis*	9	14.54	2.28	11.0	16.7	
*T. denticola*	9	16.06	3.13	11.8	20.5	

*n* = 9 denotes three biological replicates analyzed in triplicate.

* Statistically significant.

A comparison of the mean ZOI against different organisms between *P. niruri* and the triple antibiotic paste is presented in Table 2.

**Table 2:** Comparison of the mean zone of inhibition against different organisms between *P. niruri* and the triple antibiotic paste using the independent Student’s *t*-test.

Organism	Group	*N*	Mean	SD	Mean Diff	*p*-Value
*E. faecalis*	*P. niruri*	9	10.10	0.71	−54.57	<0.001*
	Triple antibiotic paste	9	64.67	0.87		
*P. gingivalis*	*P. niruri*	9	14.54	2.28	−27.32	<0.001*
	Triple antibiotic paste	9	41.87	0.43		
*T. denticola*	*P. niruri*	9	16.06	3.13	−46.06	<0.001*
	Triple antibiotic paste	9	62.11	1.62		

* Statistically significant.

For *E. faecalis*, *P. niruri* showed a mean ZOI of 10.10 ± 0.71 mm, whereas the TAP demonstrated a much higher mean ZOI of 64.67 ± 0.87 mm, with a mean difference of −54.57 mm, which was statistically significant at *p* < 0.001.

For *P. gingivalis*, *P. niruri* showed a mean ZOI of 14.54 ± 2.28 mm, while the TAP exhibited a significantly higher mean ZOI of 41.87 ± 0.43 mm, with a mean difference of −27.32 mm, which was statistically significant at *p* < 0.001.

For *T. denticola*, *P. niruri* showed a mean ZOI of 16.06 ± 3.13 mm, whereas the TAP displayed a much higher mean ZOI of 62.11 ± 1.62 mm, with a mean difference of −46.06 mm, which was statistically significant at *p* < 0.001.

This analysis highlights the superior antibacterial efficacy of the TAP over *P. niruri* across all the tested organisms, with the greatest difference observed for *E. faecalis*.

The MIC and MBC values of the *P. niruri* ethanolic extract against the tested microorganisms are summarized in Table 3. Among the three pathogens, *P. gingivalis* demonstrated the lowest MIC (3.91 mg/mL), indicating the highest susceptibility to the extract, followed by *T. denticola* (7.81 mg/mL) and *E.s faecalis* (31.25 mg/mL).

**Table 3:** MIC and MBC of *P. niruri* against all three microorganisms.

Tested microbe	MIC (mg/mL)	MBC (mg/mL)	MBC/MIC ratio
*E. faecalis*	31.25	125.00	4
*P. gingivalis*	3.91	125.00	31.9
*T. denticola*	7.81	250	32

The MBC values were considerably higher than the corresponding MICs for all microorganisms, resulting in MBC/MIC ratios of 4 for *E. faecalis*, 31.9 for *P. gingivalis*, and 32 for *T. denticola*. These ratios suggest that the extract had a predominantly bacteriostatic effect rather than a bactericidal action, particularly against the obligate anaerobes. This interpretation is further supported by the ultrastructural alterations observed in SEM and TEM analyses, which revealed extensive membrane disruption without complete cellular eradication.

**Figure 2:**
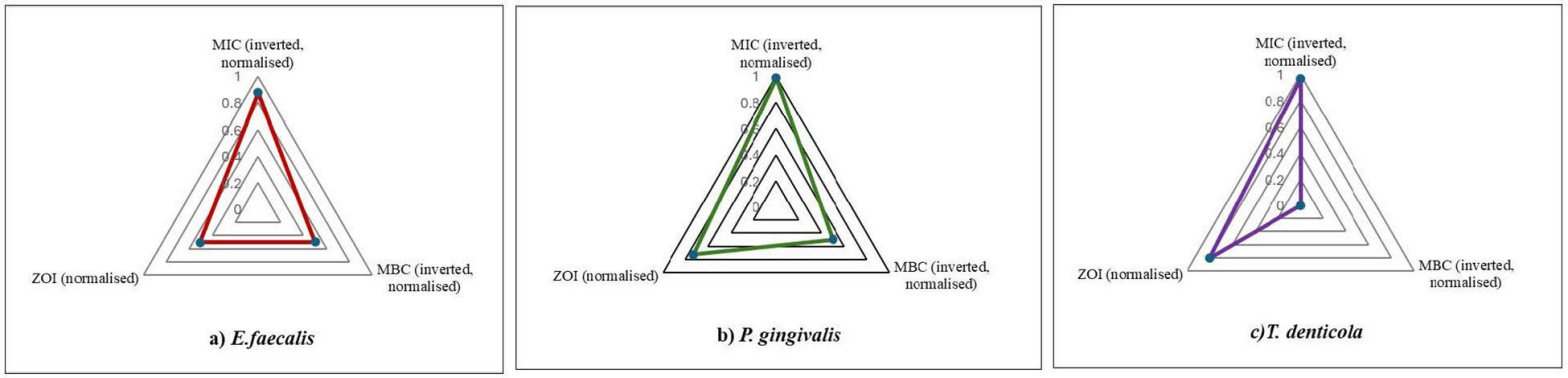
**(a–c)** Radar plots that summarize the relative antimicrobial activity of *P. niruri* against **(a)** *E. faecalis*, **(b)** *P. gingivalis*, and **(c)** *T. denticola* based on the ZOI, MIC, and MBC. The MIC and MBC values were inversely scaled and normalized to a 0–1 range prior to graphical representation, such that greater outward extension indicates stronger antimicrobial activity. Distinct radar profiles reflect pathogen-specific differences in inhibitory and bactericidal responses.

To facilitate an integrated visual comparison of diffusion-based inhibition, inhibitory potency, and bactericidal thresholds across the species, species-specific radar charts were constructed using normalized zone of inhibition values together with inverted and normalized MIC and MBC data (Figures 2a–c).

### 3.1 SEM and TEM results

**Figure 3:**
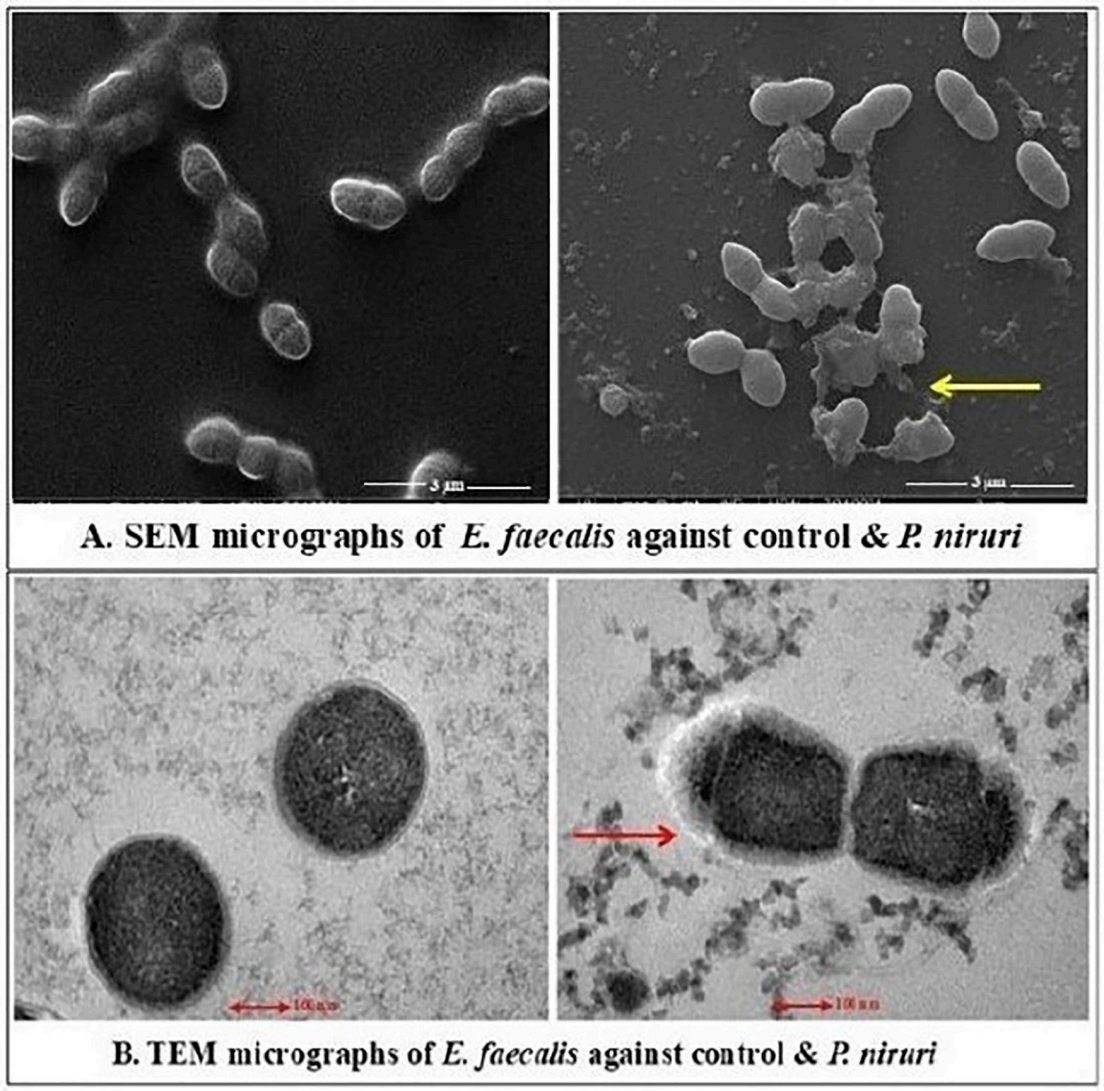
**(A)** SEM micrographs of *E. faecalis* against the control and *P. niruri.* The control group displays intact cells, with a uniform cocci morphology of *E. faecalis*. The arrow in the *P. niruri*-treated group indicates the potential lytic activity resulting in cell wall deformation, collapse, and fragmentation. **(B)** TEM micrographs of *E. faecalis* against the control and *P. niruri.* The control group exhibits well-defined cellular membranes, maintaining a coccoid morphology. The arrow in *P. niruri*-treated bacteria indicates severe membrane disruption and cytoplasmic leakage, suggesting compromised cellular integrity.

**Figure 4:**
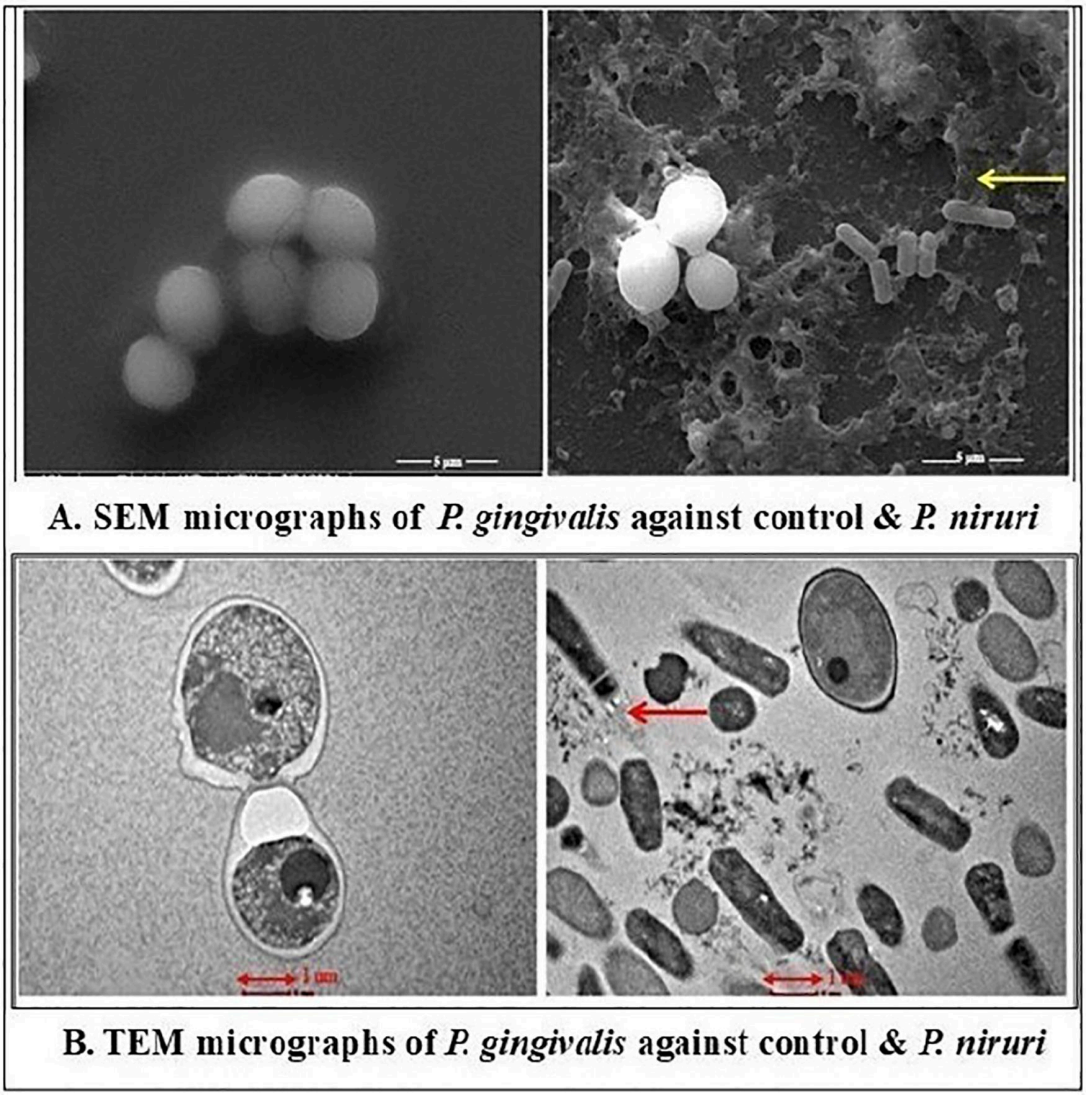
**(A)** SEM micrographs of *P. gingivalis* against the control and *P. niruri.* The control group shows closely packed cells with no visible signs of damage. The arrow in the *P. niruri*-treated group indicates evidence of bacterial cell lysis, with ruptured cell membranes and the release of cellular contents. **(B)** TEM micrographs of *P. gingivalis* against the control and *P. niruri.* In the control group, the cells exhibit intact membranes and uniform cytoplasmic density. The arrow in the *P. niruri*-treated cells indicates the disrupted cell membranes, cytoplasmic disintegration, and leakage of intracellular components.

**Figure 5:**
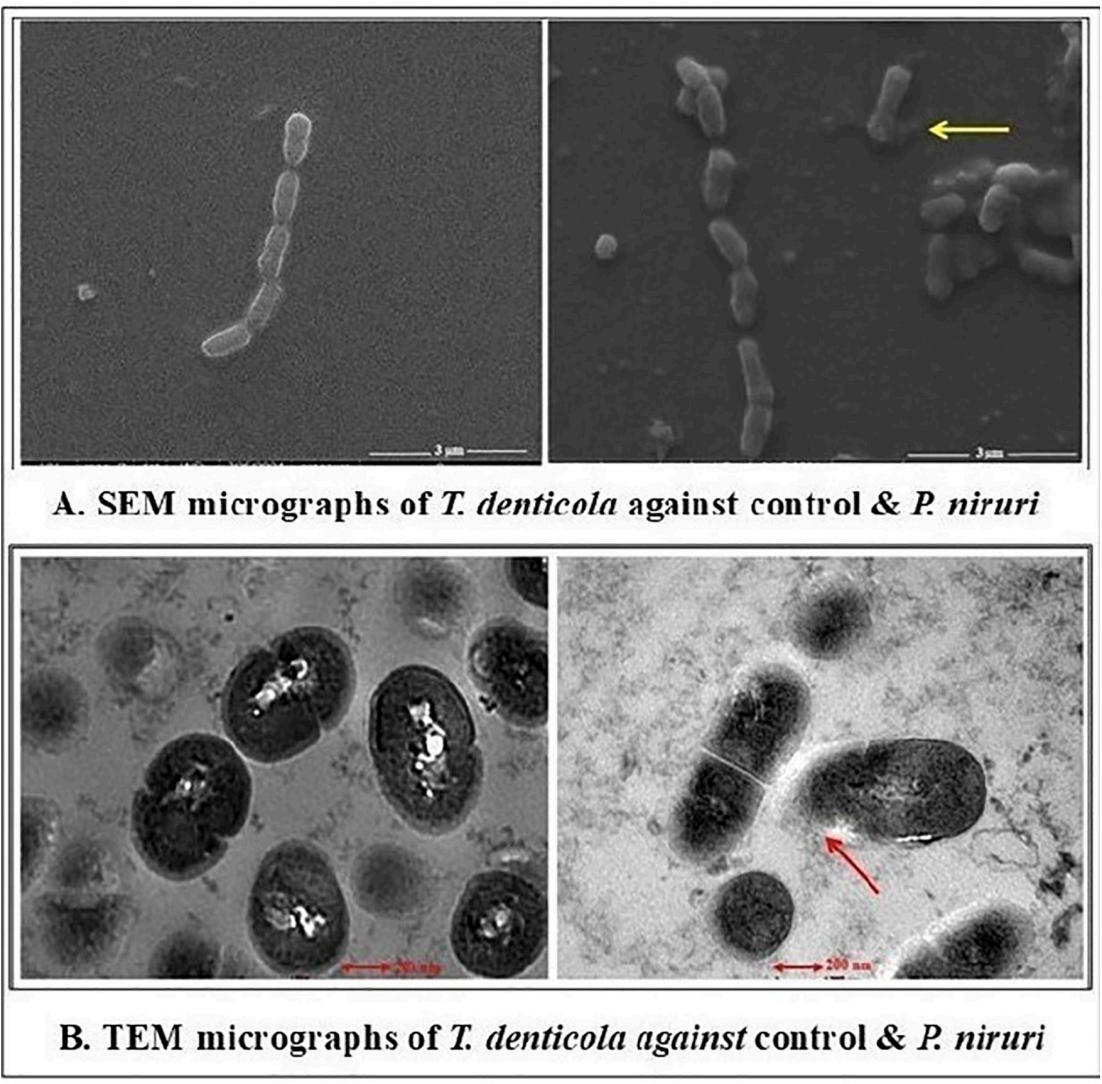
**(A)** SEM micrographs of *T. denticola* against the control and *P. niruri.* The control group shows a dense population of rod-shaped bacteria arranged in chains. The arrow in the *P. niruri*-treated group exhibits evidence of ruptured cell membranes, release of cellular contents, and a noticeable reduction in bacterial density. **(B)** TEM micrographs of *T. denticola* against the control and *P. niruri.* The control group displays uniform rod-shaped morphology of cells with intact cell membranes. The arrow in the *P. niruri*-treated group indicates altered, irregularly shaped cells with cellular swelling and possible ruptures, indicating compromised membrane integrity.

The antimicrobial effects of the *P. niruri* extract were evaluated against *E. faecalis*, *T. denticola*, and *P. gingivalis* using SEM and TEM. The extent of the structural damage varied among the tested microbes. The SEM examination of *E. faecalis* and *T. denticola* was performed at 13,000× magnification with a 3 µm particle size and in the TEM micrographs, 200 nm was the estimated size of the observed features. Whereas 5,000× magnification was used for *P. gingivalis* with a 5 µm particle size and 500 nm was the estimated size of the observed features in the TEM micrographs. *E. faecalis* displayed moderate effects, including irregular cell morphology, partial membrane disintegration, and intracellular damage (Figures 3A,B). A more pronounced antimicrobial impact was noted on *T. denticola*, where severe membrane deformation, loss of helical structure, and extensive cytoplasmic leakage were evident (Figures 4A,B). The most significant effect was observed on *P. gingivalis*, characterized by extensive surface collapse, biofilm disruption, and severe intracellular degradation, with ghost-like cells indicating loss of viability (Figures 5A,B). These findings suggest that *P. niruri* exerts a differential antimicrobial effect, with the strongest impact on *P. gingivalis*, highlighting its potential as a natural antimicrobial agent for periodontal infections.

## 4 Discussion

The results of this investigation showed that the ethanolic extract of *P. niruri* has antibacterial properties against key endodontic pathogens, including *E. faecalis*, *P. gingivalis*, and *T. denticola*. Although *P. niruri* exhibited statistically significant inhibitory effects across all the tested bacteria, its activity was markedly lower compared to the triple antibiotic paste, which served as the positive control. The mean zone of inhibition values for *P. niruri* were much lower, measuring 10.10 mm for *E. faecalis*, 14.54 mm for *P. gingivalis*, and 16.06 mm for *T. denticola*. In comparison, the TAP values were 64.67, 41.87, and 62.11 mm, respectively. These results align with previous research that demonstrated TAP's effectiveness as an intracanal medication for sterilizing necrotic root canals, particularly when applied in conjunction with LSTR procedures (11, 13).

Comparative studies have also proven TAP's higher activity compared to herbal substitutes. Despite having antibacterial qualities, the study found that *Allium sativum* extract was far less effective against *E. faecalis* than a TAP (12). In contrast, Golla et al. found that a modified TAP was more efficient against *E. faecalis* than herbal extracts (22). The same pattern was found in our investigation, where *E. faecalis* was the most resistant bacterium among the tested pathogens. The reason for its resistance could be attributed to its natural capacity to build biofilms and its endurance in extreme environmental conditions (23). Remarkably, *P. niruri* had more potent effects against obligatory anaerobes, namely *P. gingivalis* and *T. denticola*, which are often connected to periapical infections in primary teeth (5, 6, 24). *P. gingivalis* had the lowest MIC of 3.91 mg/mL and MBC of 125 mg/mL, indicating that the phytochemicals in the plant extract may be more effective at disrupting anaerobic bacterial cell walls. SEM and TEM investigations confirmed this, indicating significant structural damage in *P. gingivalis*, including membrane breakdown and loss of integrity. These findings are consistent with the structural abnormalities observed by Yun et al. in *P. gingivalis* along with other microbial species exposed to combined herbal extracts (25). Despite its promising properties, *P. niruri* has a lower antibacterial impact than TAP. The high MBC/MIC ratios of 31.9 and 32 for *P. gingivalis* and *T. denticola*, respectively, indicate its bacteriostatic rather than bactericidal activity (Table 3). The radar profiles further illustrate species-specific antimicrobial behavior, with *P. gingivalis* displaying consistently high relative values across inhibitory and bactericidal parameters, whereas *E. faecalis* exhibited a restricted profile reflecting reduced susceptibility. *T. denticola* showed an intermediate pattern characterized by lower bactericidal thresholds relative to inhibitory effects, consistent with the observed MBC/MIC ratios. These integrated antimicrobial profiles are further supported by ultrastructural observations. Species exhibiting higher relative inhibitory and bactericidal profiles in the radar analysis, particularly *P. gingivalis*, showed more extensive membrane disruption and intracellular degradation under SEM and TEM, whereas *E. faecalis*, which displayed a restricted radar profile, demonstrated comparatively moderate structural alterations. This could explain the necessity of its higher concentration in severe cases for total bacterial eradication. In another investigation, together with *P. gingivalis*, it displayed substantial antibacterial action against *Aggregatibacter* *actinomycetemcomitans* (26). From a clinical perspective, the antimicrobial profile of *P. niruri* suggests potential applicability as an adjunctive or alternative intracanal medicament, particularly in pediatric endodontics, where concerns related to antibiotic resistance, tooth discoloration, and systemic exposure limit the prolonged use of conventional antibiotic combinations such as a triple antibiotic paste (13). Although the TAP demonstrated superior bactericidal efficacy, the predominantly bacteriostatic activity of *P. niruri*, as reflected by the elevated MBC/MIC ratios, may still be clinically relevant in suppressing microbial proliferation and virulence when combined with mechanical debridement and irrigation procedures. Moreover, the pronounced ultrastructural damage observed in obligate anaerobes supports its potential role in targeting pathogens associated with persistent periapical infections, thereby contributing to improved disinfection outcomes while minimizing adverse effects associated with synthetic antimicrobials. Previous cytotoxicity studies have also reported favorable safety and biocompatibility profiles for *P. niruri* extracts for oral applications, further supporting its translational potential (17).

A significant contribution of this research is that, to date, no study has been undertaken to assess the antibacterial activity of *P. niruri* against *T. denticola*. This constitutes a substantial research gap, particularly in periodontal and endodontic infections, where *T. denticola* is a key constituent of the oral microbiome, causing persistent inflammation, tissue degradation, and, eventually, tooth loss if left untreated (27).

In this research, ethanol, a polar solvent, was used in the extraction process to efficiently extract a wide range of polar and non-polar molecules from the leaves. It is particularly successful in extracting bioactive chemicals such as phenolic compounds, lipids and fatty acids, and terpenoids, with the advantage of being safe for use in pharmaceuticals (28). The ethanolic extract of *P. niruri* demonstrated a mechanism of action on both Gram-positive (*E. faecalis)* and Gram-negative bacteria (*P. gingivalis* and *T. denticola)*. A possible explanation is that the outer lipids in the external membrane of Gram-negative microbes are distributed irregularly, comprising lipopolysaccharide and phospholipids. This acts as a barrier to many environmental elements, including antibiotics. Hence, they are thought to be more resistant. In contrast, this pattern is absent in Gram-positive bacteria. Furthermore, lipotheichoic acids, which are distinctive and vital structural elements in the cell walls of Gram-positive bacteria, may serve as suitable treatment targets for *P. niruri's* bioactive compounds. This could be a better justification for its mechanism of action on different types of microorganisms. The plant's ability to combat both Gram-positive and Gram-negative bacteria may indicate that it contains broad-spectrum medicinal components (29).

Based on these previous findings and our study results, we can conclude that the *P. niruri* leaf extract is a promising agent against root canal pathogens. Although Gram-negative bacteria are generally known for their robust outer membrane acting as a formidable barrier against many antimicrobial agents, including plant extracts, this study's SEM and TEM evaluations provide clear visual evidence that the *P. niruri* extract exerted significant structural damage and had a pronounced antimicrobial impact on the Gram-negative pathogens *P. gingivalis* and *T. denticola*. The antibacterial activity of *P. niruri* against *T. denticola* is conceptually attributed to phytochemical-mediated disruption of the bacterial cell membrane. The extract has a unique phytochemical composition, including the presence of triterpenoids, glycosides, alkaloids, saponins, tannins, flavonoids such as astragalin, and lignans such as phyllanthin and hypophyllanthin that possess amphipathic properties, enabling interaction with membrane lipids and membrane-associated proteins (29). Such interactions are known to alter membrane permeability, destabilize membrane structure, and impair essential functions, including nutrient transport and the maintenance of electrochemical gradients (30). In this study, SEM revealed membrane deformation and surface irregularities in treated *T. denticola*, while TEM provided direct evidence of disrupted membrane boundaries and cytoplasmic leakage, supporting a membrane-targeted mechanism. The absence of rapid cell lysis and the elevated MBC/MIC ratios suggest a predominantly bacteriostatic mode of action consistent with membrane destabilization rather than complete bacterial eradication (31).

*P. niruri*, a medicinal plant noted for its broad-spectrum pharmacological effects, may be a feasible natural option, considering the concerns about developing antibiotic resistance. The development of innovative, plant-based oral healthcare products, particularly for the more sustainable and biocompatible treatment of dental infections, may benefit from further research into its effectiveness against *T. denticola*.

The limitations of this study include the fact that it did not use a variety of extract solvents at varied concentrations and only examined ethanolic extracts of the herb. This was an *in vitro* study; as the *in vivo* environment is different, we cannot expect the same findings when using the herb in *in vivo* conditions. This study only utilized the herbs' leaves; different parts of the plant, such as the seeds, stem, and fruits, should be investigated for their antibacterial properties. In addition, an extensive phytochemical analysis could enhance our understanding of its antibacterial activity. Future studies should demonstrate the beneficial effects of the plant components by using variables including pharmacokinetics, distribution, and bioavailability. The evaluation of the release profile index of the bioactive substances of the plant extract is a research gap that requires further research. This *in vitro* experimental study was a preliminary laboratory investigation that did not involve human participants, animal models, or clinical applications. Consequently, the bioavailability, long-term toxicity, and enhanced permeability-retention effects of the plant extract were not assessed, and approval from the Food and Drug Administration (FDA) was not required. Although promising antimicrobial activity was observed, future clinical research must evaluate the long-term impact, including the biocompatibility, appropriate dose, and safety characteristics of the herbal extract, compared to the currently available pulpal medicines. Additional *in vivo* research and regulatory approvals are needed prior to clinical application. For clinical trial research, a precise, standardized formulation is required to guarantee that the extract is transformed into a stable, biocompatible, and easily usable form, such as an intracanal paste, gel, irrigating solution, or liquid solution. The extract needs to be incorporated into a suitable vehicle or carrier that is stable, biocompatible, and does not compromise its antibacterial properties to guarantee the best possible delivery into the complex root canal system of deciduous teeth.

## 5 Conclusion

Based on the study’s results, a 250 mg/mL concentration of *P. niruri* could be beneficial as a potential medicament for reducing root canal pathogens. Owing to its natural source, *P. niruri* may serve as an effective alternative to the conventional chemical agents that have been employed in root canal therapy, with the added advantage of lower toxicity and fewer adverse effects. The development of plant-based therapies fosters good health and wellbeing by promoting safer and more accessible oral healthcare interventions, ultimately leading to improved patient outcomes.

## Data Availability

The original contributions presented in this study are included in the article and its Supplementary Material. Further inquiries regarding the article or Supplementary Material can be directed to the corresponding author.
